# MUC1 Vaccines, Comprised of Glycosylated or Non-Glycosylated Peptides or Tumor-Derived MUC1, Can Circumvent Immunoediting to Control Tumor Growth in MUC1 Transgenic Mice

**DOI:** 10.1371/journal.pone.0145920

**Published:** 2016-01-20

**Authors:** Vani Lakshminarayanan, Nitin T. Supekar, Jie Wei, Dustin B. McCurry, Amylou C. Dueck, Heidi E. Kosiorek, Priyanka P. Trivedi, Judy M. Bradley, Cathy S. Madsen, Latha B. Pathangey, Dominique B. Hoelzinger, Margreet A. Wolfert, Geert-Jan Boons, Peter A. Cohen, Sandra J. Gendler

**Affiliations:** 1 Department of Immunology, Mayo Clinic in Arizona, Scottsdale, AZ, United States of America; 2 Department of Biochemistry/Molecular Biology, Mayo Clinic in Arizona, Scottsdale, AZ, United States of America; 3 Hematology/Oncology, Mayo Clinic in Arizona, Scottsdale, AZ, United States of America; 4 Biostatistics, Mayo Clinic in Arizona, Scottsdale, AZ, United States of America; 5 Complex Carbohydrate Research Center, University of Georgia, Athens, GA, United States of America; Ohio State University, UNITED STATES

## Abstract

It remains challenging to produce decisive vaccines against MUC1, a tumor-associated antigen widely expressed by pancreas, breast and other tumors. Employing clinically relevant mouse models, we ruled out such causes as irreversible T-cell tolerance, inadequate avidity, and failure of T-cells to recognize aberrantly glycosylated tumor MUC1. Instead, every tested MUC1 preparation, even non-glycosylated synthetic 9mer peptides, induced interferon gamma-producing CD4^+^ and CD8^+^ T-cells that recognized glycosylated variants including tumor-associated MUC1. Vaccination with synthetic peptides conferred protection as long as vaccination was repeated post tumor challenge. Failure to revaccinate post challenge was associated with down-regulated tumor MUC1 and MHC molecules. Surprisingly, direct admixture of MUC1-expressing tumor with MUC1-hyperimmune T-cells could not prevent tumor outgrowth or MUC1 immunoediting, whereas *ex vivo* activation of the hyperimmune T-cells prior to tumor admixture rendered them curative. Therefore, surrogate T-cell preactivation outside the tumor bed, either in culture or by repetitive vaccination, can overcome tumor escape.

## Introduction

Novel immunomodulatory treatments such as checkpoint inhibitors have revealed that many types of human cancer induce endogenous immune responses which can be disinhibited to result in tumor rejection [1]. However, most cancer patients are incompletely responsive or unresponsive to such immunomodulation, likely reflecting the absence of a serviceable endogenous immune response [2,3]. These refractory cancers may require additional strategies such as vaccination to tumor antigens to become responsive to immunomodulators [4].

MUC1 is an attractive antigen for this purpose, due to its high level of expression by the majority of human cancers and its reported immunogenicity [5,6]. MUC1 is a cell-associated mucin largely consisting of tandem repeats (TR), sea urchin sperm protein, enterokinase and agrin (SEA), transmembrane and cytoplasmic tail (CT) domains [7]. The CT acts as a scaffold for multiple signaling pathways and the full-length protein is oncogenic [8,9,10,11]. MUC1 is heavily glycosylated, with more than half its large molecular mass attributable to carbohydrates. The *O*-linked carbohydrates are attached to MUC1 at the five serines and threonines within each TR. Glycosylation patterns vary in each tissue and physiological condition. Furthermore, it has been widely demonstrated that cancer gives rise to distinctive, aberrant MUC1-associated glycosylation, likely due to mutations in the Cosmc chaperone for T-synthase (core 1 β3-galactosyltransferase), increased sialylation and/or deregulation of glycosyltransferase genes. This generates truncated carbohydrate structures such as Tn (αGalNAc-Thr/Ser) and STn (αNeu5Ac-(2, 6)-αGalNAc-Thr/Ser), which are not normally expressed in peripheral tissues [12,13].

While individual MUC1 vaccine clinical trials have shown promise, the therapeutic effects are often sub-optimal or require subset analysis to demonstrate efficacy [14,15,16,17,18,19,20,21,22,23,24]. For example, a lipid-encapsulated 25 amino acid non-glycosylated peptide (BLP25, Tecemotide) derived from the MUC1 TR demonstrated a convincing 10-month survival advantage within an 806 patient subset who received concurrent chemoradiotherapy for regionally advanced non-small cell lung cancer [14]. Further evidence of activity in conjunction with chemotherapy was seen in another advanced non-small cell lung cancer trial utilizing the TG4010 vaccine, a recombinant virus Vaccinia Ankara encoding both MUC1 and IL-2 [18]. In multiple tumor types, a phase I trial of ONT-10, an aberrantly glycosylated 2TR peptide, demonstrated disease stabilization in 65% of patients with advanced disease [22]. A pilot Phase III trial of oxidized mannan-MUC1 (5 TR) in stage II breast cancer patients reported a recurrence rate of 12.5% (2 of 16) in patients receiving mannan-MUC1 vs. 60% (9 of 15) for placebo in a 15-year follow-up [20]. These results imply that multiple formulations of both non-glycosylated and glycosylated MUC1 vaccines derived from the TR may engender therapeutic effects. Therapeutic activity (disease stabilization in most patients) was also seen in a small pilot trial that employed vaccine moieties outside the TR in multiple myeloma patients [23]. Additional maneuvers, such as transfer of cytotoxic T lymphocytes (CTLs) primed by a MUC1-expressing pancreatic cell line and dendritic cells (DCs) pulsed with a 100mer (5 TR) synthetic peptide, demonstrated disease stabilization (n = 5) and one 5-year complete response with elimination of multiple lung metastases in a clinical trial (n = 20) for stage III and IV pancreatic cancer patients [24].

Anti-tumor responses have been observed in various preclinical mouse models, although many different vaccine preparations, adjuvants, and methods of administration make it difficult to compare results. The ability of MUC1 based vaccines to protect against MUC1-expressing tumor in either wild type or MUC1.Tg mice have shown a range of effects [25,26,27,28,29,30]. The frequent inability to generate definitive tumor protections has sometimes been associated with excessive T regulatory activity [31,32]. Some tumor models such as B16.MUC1 and MC38.MUC1 have proven to be preventable by MUC1 vaccines [29,33]. Models such as MC38.MUC1 appear to require prolongation of vaccination into the post challenge period [29,34]. Our own lab has frequently observed the capacity of vaccination to significantly slow tumor progression [30,35,36,37].

Many questions remain as to how best to deploy MUC1 as a tumor vaccine. As a self-antigen, recognition of individual MUC1 epitopes may be diminished by prior T-cell tolerance and thymic deletion [38,39]. This may favor recognition of MUC1 neoantigens that were not exposed prior to the tumor-bearing state [40]. However, such tumor-associated glycoforms display tremendous variation from patient to patient as well as within individual patients, rendering it possible that vaccines may not target epitopes therapeutically relevant to many patients [6,12].

We explored these issues in a mouse model transgenic for human MUC1 which simulates therapeutic issues such as T-cell tolerance, autoimmunity, and heterogeneity of aberrant glycosylation among relevant tumor models [27,41]. We compared the physiologic and therapeutic consequences of deploying different MUC1 antigenic preparations such as non-glycosylated 9mers, O-glycosylated 9mers mimicking aberrant glycosylation, longer peptides containing class I and class II epitopes, and rotating lysates of naturally glycosylated MUC1-expressing tumor lines. Our studies demonstrated that a diverse array of MUC1 antigen preparations can be deployed effectively as a vaccine, but even the most effective preparations are vulnerable to issues of tumor immunoediting.

## Results

### A wide array of MUC1 antigen preparations are effective for generating specific immune responses in tolerant MUC1.Tg and non-tolerant mice

The efficacy of MUC1 peptides to develop MUC1-specific CD4^+^ and CD8^+^ T-cell responses was examined in MUC1.Tg and C57BL/6 wild type (WT) mice. The transgenic mice express the human MUC1 transgene in a histological pattern consistent with that observed in humans. Hence, MUC1.Tg mice are an appropriate model for investigating immunity and mechanisms of central and peripheral tolerance to the tumor antigen MUC1 as well as such phenomena as epitope spreading [41].

**Fig 1:**
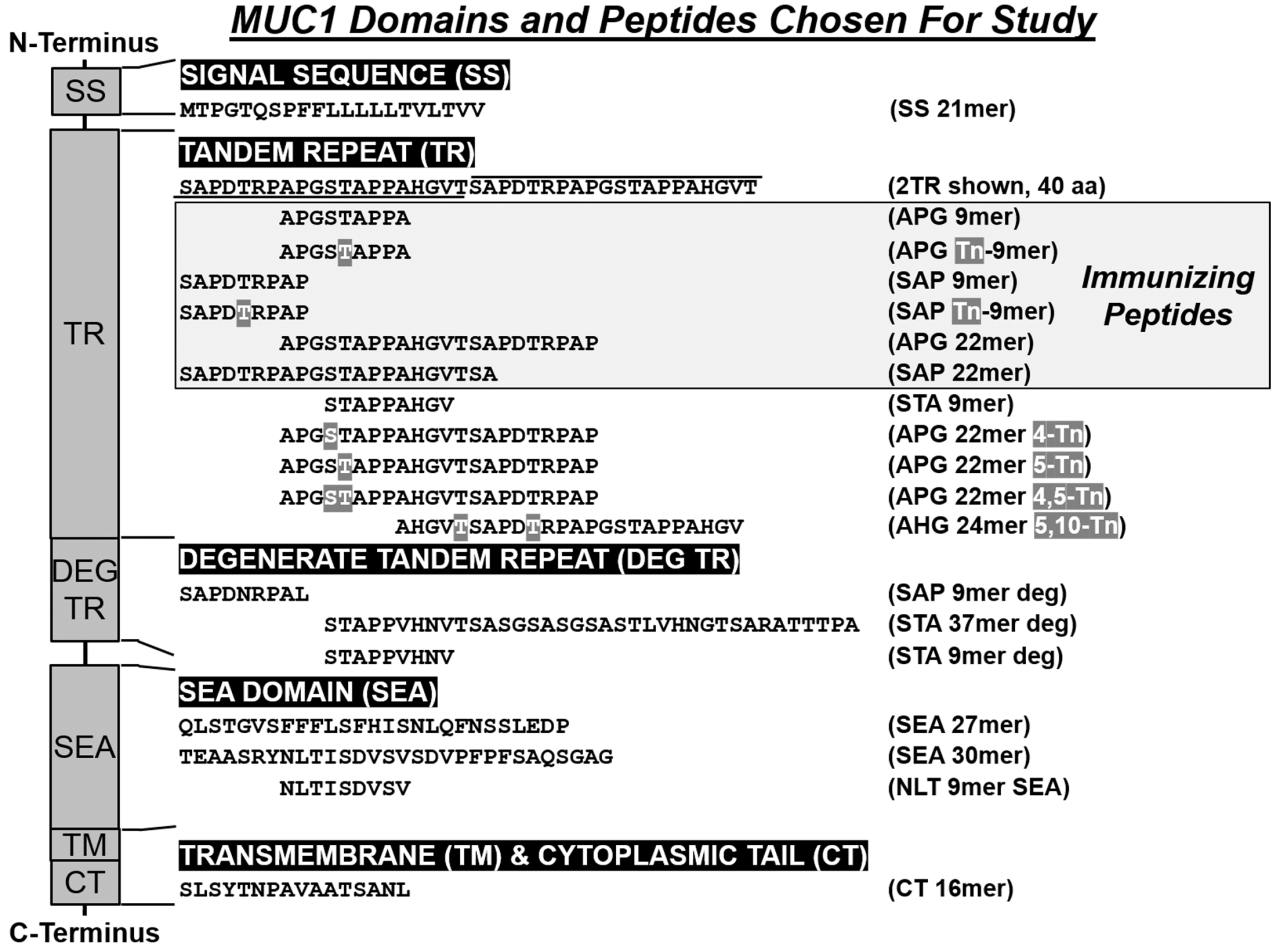
Diagram of MUC1 Domains and Peptides Chosen for Study. The domains of MUC1 are shown on left side of the diagram with the sequences studied listed below the domains and the peptide name on the right side. Immunizing peptides and serines (S) and threonines (T) that are *O*-glycosylated with N-Acetylgalactosamine (Tn) are shaded.

**Fig 2:**
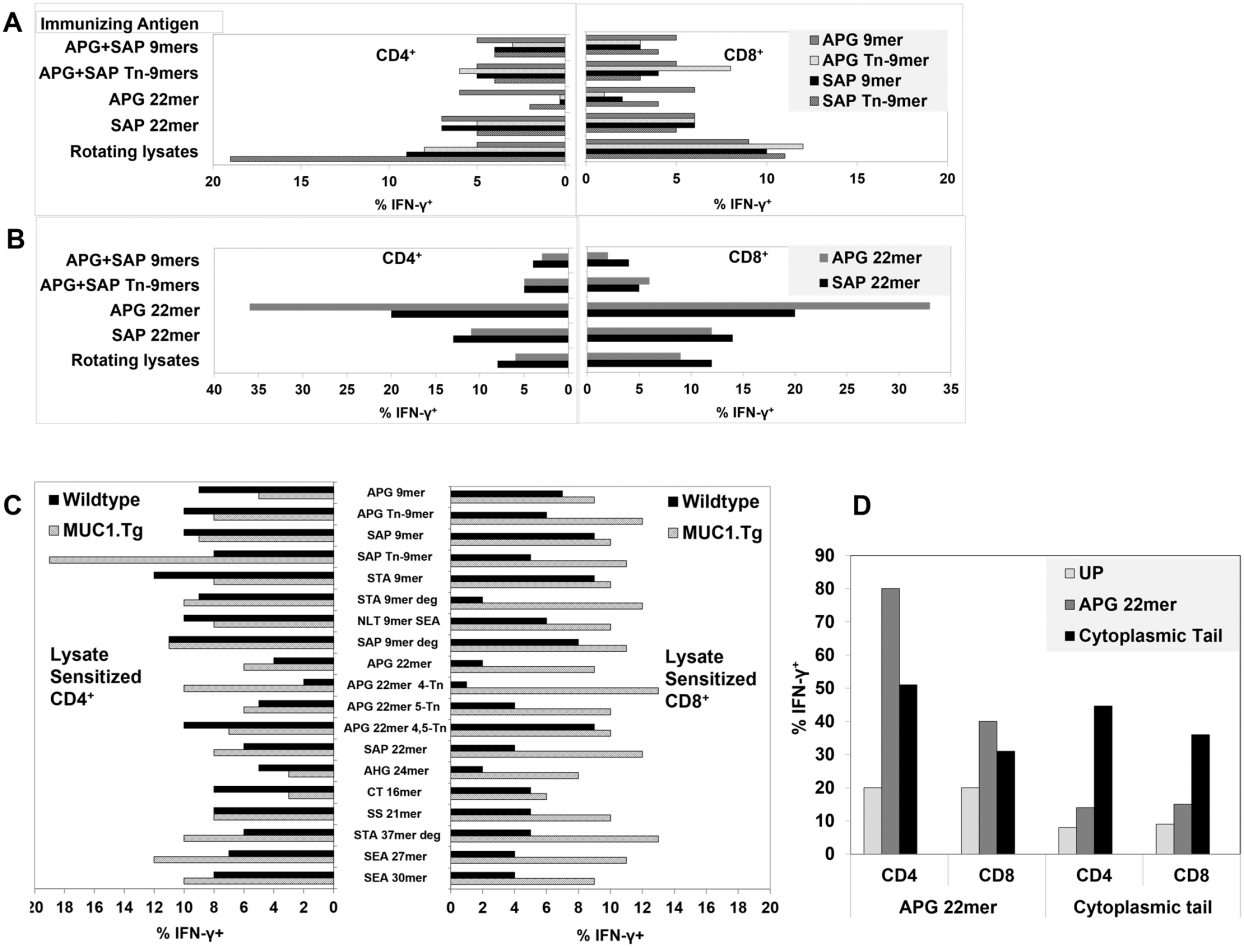
Diverse MUC1 Antigen Preparations Generate Specific Immune Responses in Tolerant MUC1.Tg and Non-Tolerant WT Mice. MUC1.Tg mice were given three immunizations with vaccines containing 9mers, 22mers or rotating tumor lysates (Fig 1). Lymph node-derived T-cells were culture expanded for 7–14 days with DCs pulsed with the immunizing antigens. Antigen-specific T-cells were enumerated for intracellular IFN-γ production when re-stimulated with DCs pulsed with short peptides **(A)** and long peptides **(B)**. Data are shown after subtracting background from unpulsed DCs to facilitate visual comparisons. See Fig 2D for examples of representative unsubtracted backgrounds. A representative of 2 experiments is shown; pools of 7 mice were used. **(C) Wide specificity of the lysate-sensitized T-cells**: Lysate sensitized T-cells from MUC1.Tg mice or WT mice showed specificity against 19 out of 19 MUC1 peptides from both TR and non-TR regions. **(D)** MUC1.Tg mice were immunized twice with vaccines containing either long peptides from TR, APG 22mer (APGSTAPPAHGVTSAPDTRPAP) or the CT peptide (SLSYTNPAVAATSANL). After *in vitro* stimulation with DCs pulsed with immunizing peptides, antigen specific T-cells were analyzed for intracellular IFN-γ against dendritic cells pulsed with peptides (APG 22mer or CT) or no peptide (UP). One representative of three experiments is shown; pools of 7 mice were used in each experiment.

We compared vaccinations with various synthetic peptides derived from the TR sequence (established K^b^, D^b^ epitopes) including short peptides (glycosylated vs non-glycosylated 9mers) and long (22mer) peptides (Fig 1). We also tested rotating lysates which contained naturally glycosylated full length MUC1. Lysates from different cell lines were used to drive the response toward MUC1 and to include the diverse glycoforms of MUC1 on tumors originating from different tissue types. All preparations were emulsified with CpG 1826 as adjuvant in Incomplete Freund’s Adjuvant (IFA). Three weeks after the last immunization, the draining lymph nodes were harvested and sorted. CD62L^low^ effector T-cell subsets were stimulated *in vitro* for 1–2 weeks with DCs pulsed with the immunizing peptides or, for the group immunized with rotating tumor cell lysates, with B16.MUC1 tumor lysate not used during previous *in vivo* priming. The culture-expanded T-cells were first analyzed for specific recognition of MUC1-derived synthetic peptides pulsed onto DCs. Remarkably, vaccination with any of the tested peptides or lysates was consistently able to induce strong CD4^+^ and CD8^+^ T-cell responses to the immunizing antigen, even in tolerant MUC1.Tg mice (Fig 2). Furthermore, immunizations with either non-glycosylated or glycosylated peptides resulted in generation of MUC1-specific T-cells that recognized both naked and glycosylated MUC1 antigen, due either to epitope spreading or to cross-recognition.

In addition to such diversification, vaccinations even to 9mers were sufficient to generate CD4^+^, in addition to CD8^+^ specific T-cell responses as reported previously [42,43]. Alternatively, mice vaccinated with 22mers gave rise not only to T-cells that strongly recognized the long peptides but also some, but not all, of the embedded 9mers (Fig 2A and 2B). Superior presentation of embedded peptides was observed with rotating tumor cell-derived lysates rather than synthetic peptides (Fig 2A–2C). Also distinctive to lysate-sensitized T-cells, 19% of CD4^+^ T-cells recognized SAP Tn-9mer but only 9% recognized SAP 9mer, consistent with selective reactivity with the glycoform. In addition, lysate-sensitized T-cells recognized 19 out of 19 additional tandem repeat as well as non-tandem repeat MUC1 peptides (Fig 2C). Unexpectedly, even though peptide vaccinations were confined to the TR region of MUC1, they gave rise to T-cells which also recognized the CT region, even though the latter was absent from the vaccine. Such sensitization demonstrated true intramolecular epitope spreading. Similarly, we observed that vaccination with a CT peptide gave rise to recognition of a TR sequence that was not part of the peptide vaccine (Fig 2D).

When WT instead of MUC1.Tg mice were vaccinated with rotating lysates, an equally diverse repertoire developed. Importantly, however, the proportion of recognized epitopes by both CD4^+^ and CD8^+^ T-cells was quite different between MUC1.Tg and WT mice. This most likely represented skewing of the repertoire in the transgenic mice due to life-long exposure to MUC1 as a self-antigen (Fig 2C) [44]. Peptide dose titrations demonstrated that, despite any prior repertoire modulation, MUC1-specific T-cells obtained from WT or MUC1.Tg mice were maximally avid even without exposure to avidity-enhancing IL-12 (S1 Fig) [45].

### T-Cells from MUC1-Vaccinated Mice Recognize Tumor-Associated MUC1

**Fig 3:**
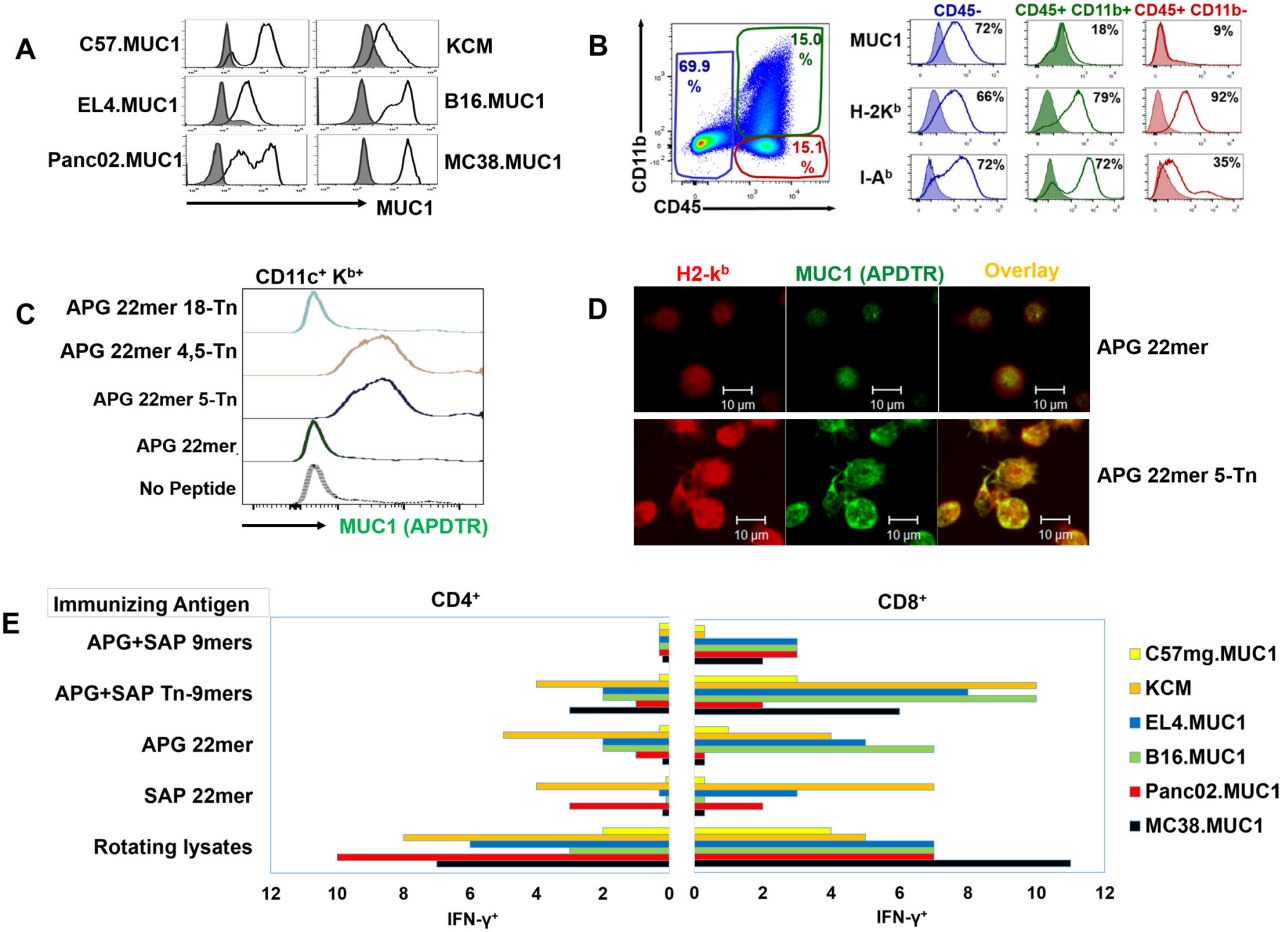
Aspects of Antigen Presentation Relevant to Tumor Recognition are Shown. **(A)** Tumor cell expression of MUC1 was stable *in vitro* (C57mg.MUC1: 86%; KCM: 73%; EL4.MUC1: 79%; B16.MUC1 and Panc02.MUC1: 95%; MC38.MUC1: 99%) **(B)** Irradiated tumor digests passed in syngeneic mice prior to processing were used to assess T-cell recognition of tumor-associated MUC1. Representative flow data for Panc02.MUC1 is shown. MUC1 was predominantly expressed on the tumor cell population, while both MHC class I and II molecules were expressed by CD45^+^ host cells as well as tumor cells. **(C) Glycosylation alters DC processing of MUC1**. DCs were pulsed overnight with non-glycosylated APG 22mer or one of three glycoforms of APG 22mer (5-Tn; 4, 5-Tn or 18-Tn). The DCs were then analyzed for presentation of the C-terminal peptide SAPDTRPAP (PDTR) by flow cytometry with anti-MUC1 (BC2-Alexa488) specific for PDTR. Cells staining positively for PDTR also co-stained for CD11c^+^, K^b^ (Fig 3C) and I-A^b^ (not shown). Detection of PDTR on DCs was only observed if APG 22mer was glycosylated at 4-Tn or 4, 5-Tn (Fig 3C). **(D) Glycosylation promotes co-localization of SAPDTRPAP with class I molecules**. DCs in chamber slides were stimulated overnight with either non-glycosylated (APG 22mer) or glycosylated (APG 22mer 5-Tn) peptides and stained with anti-MUC1 (BC2-Alexa488; green) and anti-H-2K^b^ followed by secondary goat anti-mouse IgG-labeled-Alexa633 (red). Representative confocal images showed stronger co-localization (yellow) of epitope SAPDTRPAP with H-2K^b^ on the DCs pulsed with the glycopeptide. The experiment was repeated two times. **(E) Individual forms of antigen during vaccination elicited differential recognition of MUC1-expressing tumors**. Effector T-cells from MUC1.Tg mice immunized with vaccines containing either 9mers, 22mers or rotating tumor lysates were stimulated *in vitro* for 7–14 days with DCs pulsed with immunizing peptides or B16.MUC1-expressing tumor cell lysate. The stimulated T-cells were co-cultured with various MUC1-expressing or MUC1 non-expressing irradiated tumor digests (C57mg.MUC1, C57 WT; KCM, KCKO; EL4.MUC1, EL4.WT; B16.MUC1, B16.neo; Panc02.MUC1, Panc02.neo and MC38.MUC1, MC38.neo) and stained for intracellular IFN-γ. Data showed MUC1-specific responses that were determined by subtraction of background reactivity of the corresponding MUC1 non-expressing tumor digests. Representative data of two independent experiments are shown; pools of 7 mice were used.

We next investigated whether T-cells from mice vaccinated with different MUC1 preparations recognized tumor-associated MUC1 as well as synthetic peptides. We prepared whole cell digests from tumor-bearing mice that contained tumor cells as well as host antigen presenting cells, each of which possessed the MHC machinery to present tumor-associated MUC1. All tumor lines which expressed MUC1 *in vitro* prior to inoculation (Fig 3A) continued to express MUC1 in the tumor digests (Fig 3B).

In examining the ability of T-cells to recognize diversely glycosylated tumor-associated MUC1, it was important to consider the role that glycosylation played in antigen processing. Based on previous observations by Hanisch [46], it was likely that glycosylation of serine or threonine residues would displace normal cleavage sites, resulting in liberation of unique embedded epitopes from glycosylated MUC1. To test this we employed antibodies to MUC1 that can recognize epitopes from the TR, either endogenously expressed or bound to MHC molecules [47]. Consistent with our hypothesis, the embedded 9mer SAPDTRPAP (H-2K^b^-restricted) was not effectively presented when DCs were fed with non-glycosylated long peptide (APG 22mer) or when glycosylation was on the T in APG 22mer 18-Tn. However, when peptides were glycosylated on serine 4, threonine 5 or both serine 4 and threonine 5, the SAPDTRPAP epitope showed coordinate expression with both class I (Fig 3C) and class II MHC molecules (not shown). Co-localization with H-2K^b^ was confirmed by confocal microsopy (Fig 3D). Therefore, glycosylated residues can affect the presentation of non-glycosylated embedded peptides.

Despite the fact that peptide- or lysate-primed T-cells achieved considerable epitope diversity during the vaccination process, it remained to be determined whether the epitopes were also expressed by tumor cells. In fact, both peptide-primed and lysate-primed T-cells proved capable of cross-recognizing whole cell MUC1-expressing tumor digests at a higher frequency than non-MUC1 expressing digests (Fig 3E).

Interestingly, priming to particular peptide or lysate preparations resulted in greater or lesser reactions to individual tumor digests. For example, T-cells primed to SAP 22mer recognized Panc02.MUC1 while minimally recognizing B16.MUC1, which was the reverse pattern observed for APG 22mer primed T-cells. We therefore proceeded to determine if recognition of tumor digests predicted effective protection against tumor challenges.

### Lysate-Sensitized but not Peptide-Sensitized Mice are Protected Against Subsequent Challenge with MUC1-Expressing Tumor

**Fig 4:**
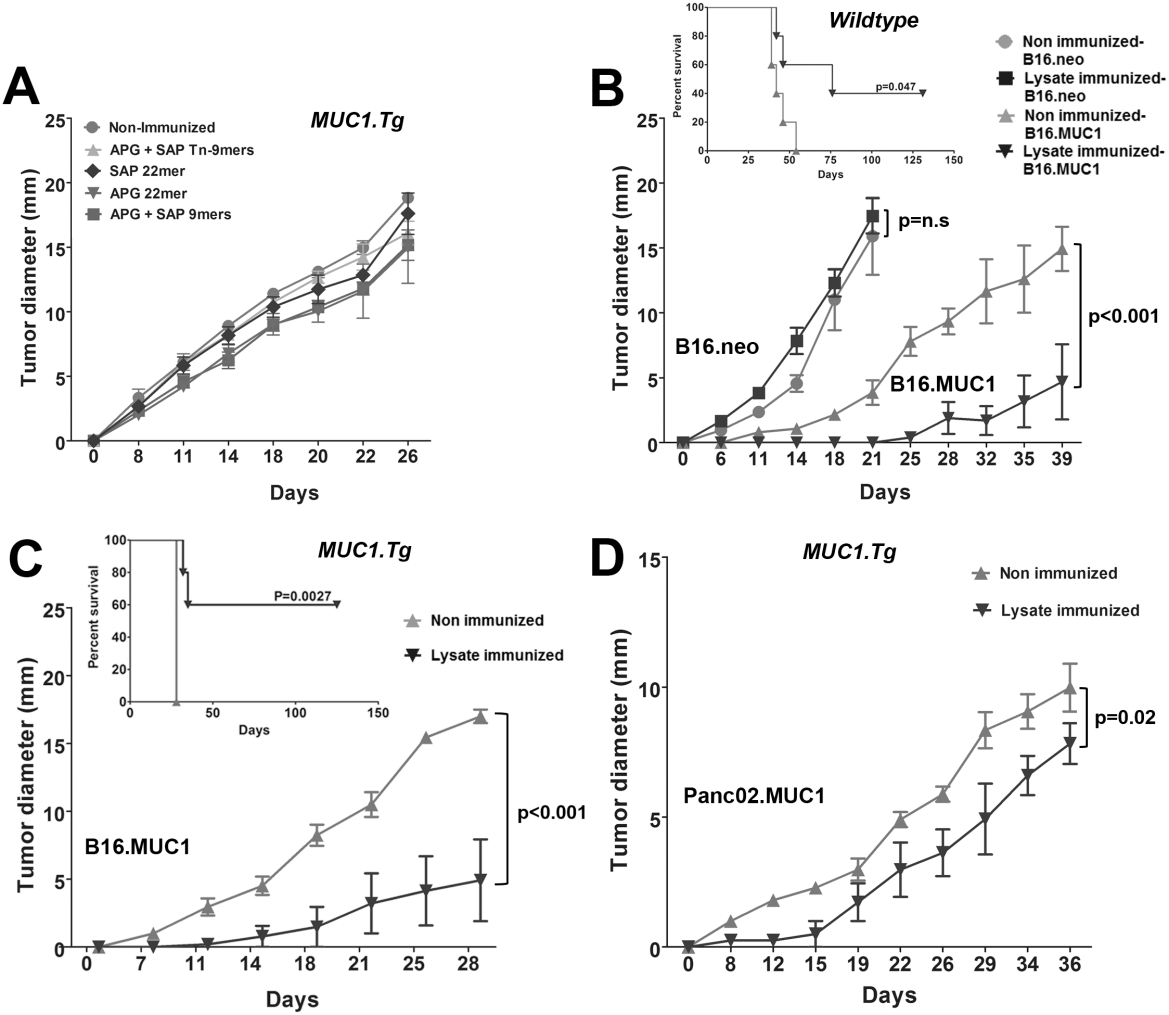
Lysate-Sensitized but not Peptide-Sensitized Mice are Protected Against Subsequent Challenge with MUC1-Expressing Tumor. MUC1.Tg or WT mice were immunized with vaccines containing rotating MUC1-expressing tumor cell lysates or peptides (Fig 1) prior to challenge with MUC1-expressing cells (B16.MUC1, Panc02.MUC1). Tumor growth was monitored by palpation. **(A)** Peptide immunizations failed to protect B16.MUC1 tumor growth; **(B) & (C)** In both WT and MUC1.Tg mice, the vaccination with rotating lysates induced MUC1-specific immune responses that cured 4 out of 10 WT mice **(B insert)** (p = 0.047) and 5 out of 10 MUC1.Tg mice **(C insert)** (p = 0.0027). B16.MUC1 tumors that grew out in both **(B)** WT and **(C)** MUC1.Tg mice showed significantly delayed tumor growth (p<0.001). The same immunizations had no effect on the B16.neo tumor outgrowth **(B**) (p = n.s). **(D)** In Panc02.MUC1 tumor challenge, the onset of tumor was significantly delayed in the immunized mice as compared to the non-immunized controls (p = 0.02). Groups of 5 mice were tested and the experiment was repeated twice.

WT or MUC1.Tg mice were immunized three or four times with either peptides or rotating lysates and then challenged with MUC1-expressing tumor cells (B16.MUC1) that were not part of prior cell lysate immunizations. Even though peptide-sensitized T-cells often recognized tumor digests robustly *in vitro*, tumor protection was never observed against B16.MUC1 challenges *in vivo* (Fig 4A). In contrast, lysate-vaccinated MUC1.Tg mice were often cured of subsequent B16.MUC1 challenge (Fig 4C and inset). Rotating lysates also effectively sensitized T-cells in WT mice to recognize and reject MUC1-expressing B16 tumors (Fig 4B and inset). When mice were challenged instead with Panc02.MUC1 (Fig 4D) or MC38.MUC1 (not shown), mice also showed significantly prolonged survival but without sustained regression. Underscoring that tumor control was a function of MUC1 recognition, rotating lysate-immunized mice were not protected against B16.neo (Fig 4B).

### Immunoediting of MUC1 and MHC Expression is Consistently Associated with Vaccine Failure

**Fig 5:**
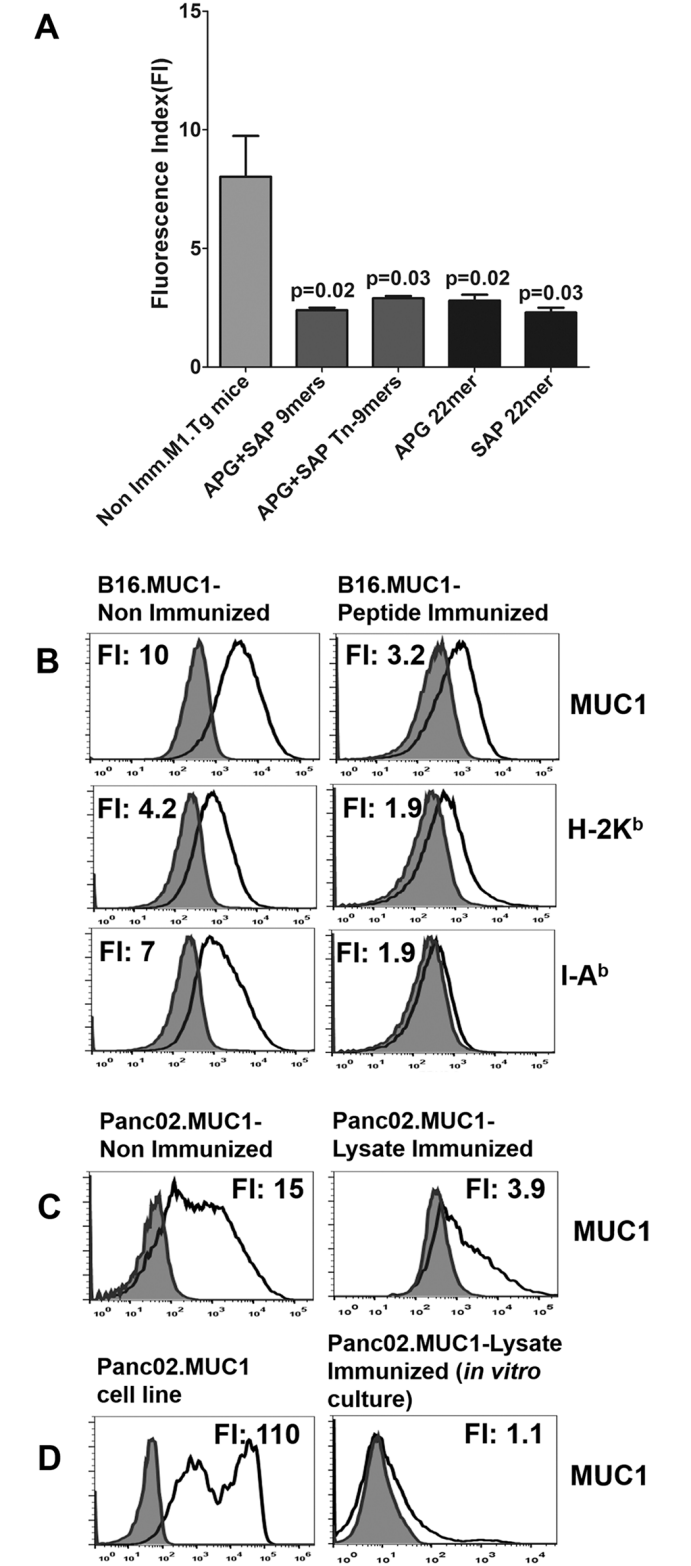
Tumor Progression Following Immunization with Either Peptides or Rotating Lysates Prior to Tumor Challenge Consistently Resulted in Decreased Expression of Tumor MUC1 and MHC Proteins. The resistant B16.MUC1 tumors that grew after peptide immunizations were excised, digested and analyzed by flow cytometry with anti-CD45 APC-Cy7, anti-MUC1 FITC (CD227), anti-H-2K^b^ PE or anti-I-A^b^ PE. Cell surface MUC1 and MHC expression were significantly decreased following peptide vaccination compared to non-immunized mice. **(A)** Each bar represents the average fluorescent index (FI) of surface MUC1 expression for each treatment group. A typical experiment with n = 3 mice per group is shown; experiments were repeated at least 3 times. **(B)** Representative histograms are shown. The peptide shown is SAP 22mer, which is representative of the results for 9mer peptides. **(C)** Similar results were seen for Panc02.MUC1 in those groups where prior lysate immunization failed to prevent tumor outgrowth. **(D)** The cell line generated from Panc02.MUC1 tumor *in vitro* in G418 maintained low MUC1 surface expression when expanded **(right panel)** as compared to the parent cell line (**D, left panel**).

Mice vaccinated to peptide prior to B16.MUC1 challenge displayed rapid tumor progression indistinguishable from unvaccinated mice (Fig 4A), but, surprisingly, also displayed down regulated MUC1 and MHC expression (Fig 5A and 5B). Such tumor escape mechanisms were also observed in the subset of B16.MUC1 challenged mice which ultimately developed progressive tumors and in Panc02.MUC1 (Fig 5A, 5B and 5C). MUC1 and MHC immunoediting were observed in both WT (not shown) and MUC1.Tg mice. In contrast to the observed down regulation of MUC1 and MHC proteins following vaccination, cell surface expression of aberrantly glycosylated moieties remained stable, suggesting that down regulation of MUC1 protein was an escape mechanism in the absence of modulated glycosylation levels (not shown). It was, therefore, evident that every tested MUC1 vaccine composition exerted a strong selection pressure, even when tumor progression rather than cure was observed.

We examined whether such immunoediting would persist phenotypically if selection pressure of the anti-MUC1 response was removed by culturing the Panc02.MUC1 tumor *ex vivo*. Surprisingly, down regulation of MUC1 expression persisted for seven passages in the complete absence of T-cell selection pressure, which was not reversed even though the cells were grown in G418 (500 ug/ml) and retained neomycin resistance (Fig 5D).

### Restarting Vaccination Post Tumor-Challenge Renders Even Peptide Vaccinations Therapeutically Active

**Fig 6:**
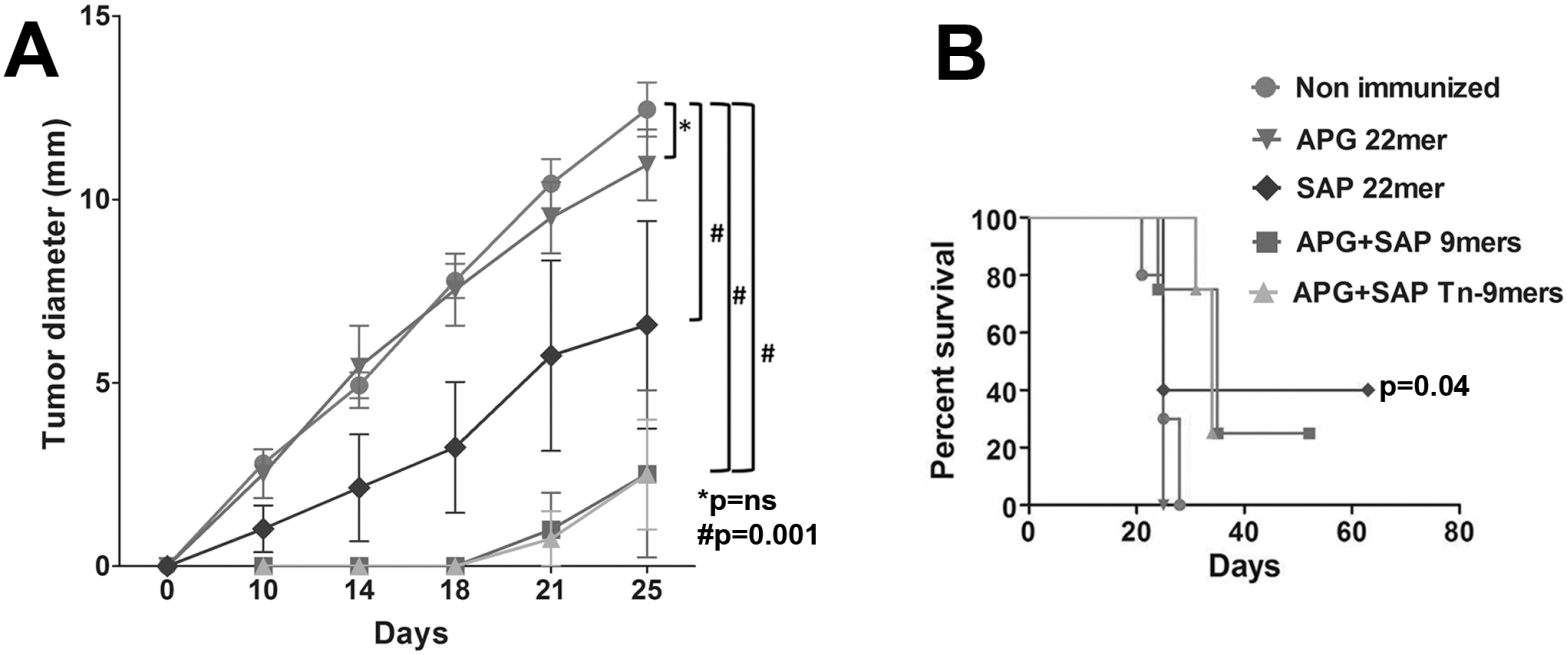
Peptide Vaccine is Rendered Therapeutically Effective if Repetitive Delivery is Continued Post Tumor Challenge. MUC1.Tg mice were given two peptide immunizations before and two after tumor challenges. Three out of four peptide vaccinations significantly delayed the growth of B16.MUC1 tumors (APG+SAP 9mer, p = 0.001; APG+SAP Tn-9mer, p = 0.001; SAP 22mer, p = 0.001) and **(B)** showed complete protection in 4 out of 10 mice (SAP 22mer) and 3 out of 10 mice (9mers and Tn-9mers), if immunizations were continued post tumor challenges. Experiment was repeated 2 times, n = 5 mice/group.

As shown above, peptide vaccination with CpG was highly effective for reversing MUC1 tolerance in MUC1.Tg mice (Fig 2), but was completely ineffectual for protecting against tumor challenges initiated after vaccinations were completed (Fig 4A). Remarkably, 3 out of 4 peptides were rendered therapeutically effective if two additional vaccines were given after the B16.MUC1 tumor challenge (Fig 6A and 6B, p = 0.001). Inoculation with CpG alone was ineffective (not shown). Both non-glycosylated and glycosylated 9mers and SAP 22mer resulted in complete eradication of tumors up to 100 days following tumor challenge with a single post-tumor challenge vaccination.

### Therapeutic Efficacy of MUC1-primed T-Cells can be Enhanced by Culture Activation

**Fig 7:**
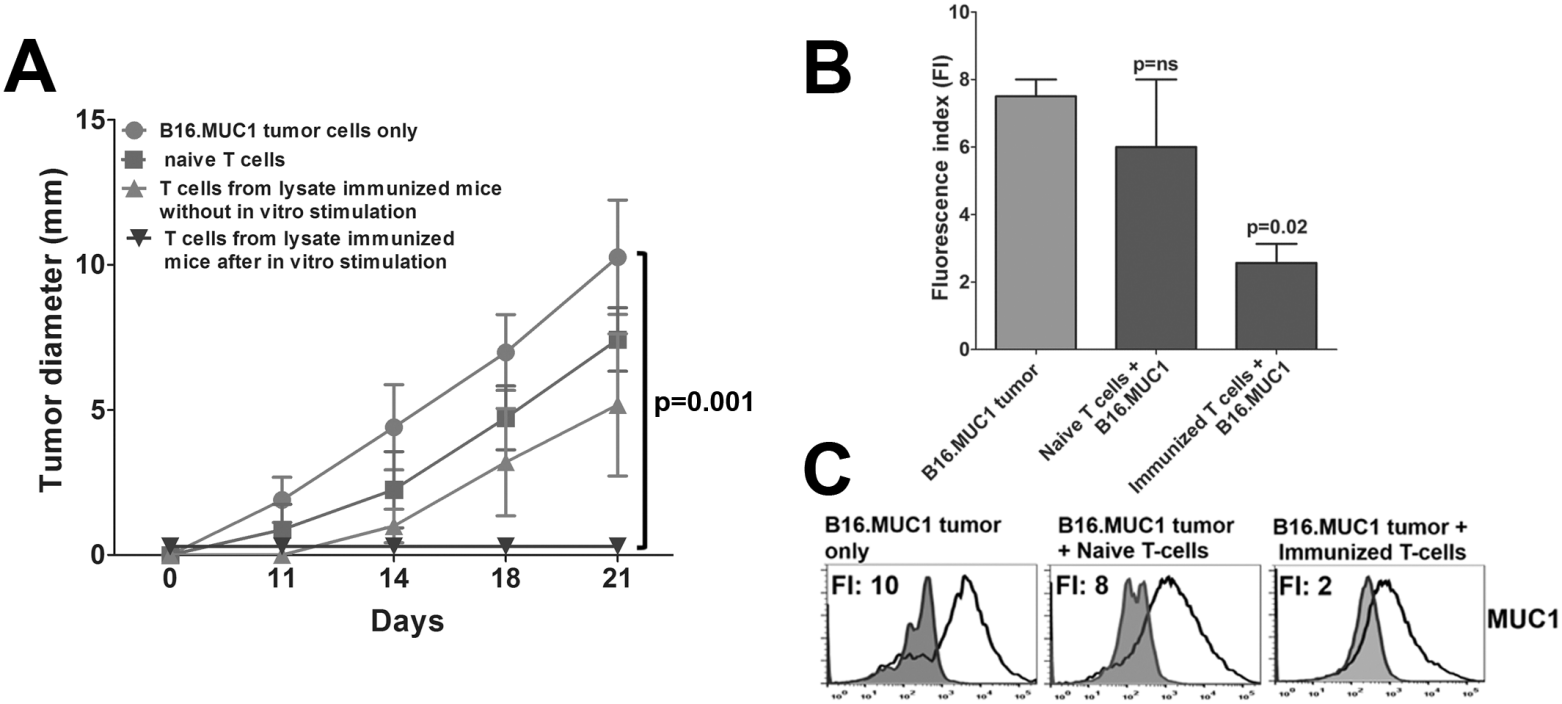
Lysate Sensitized T-Cells from MUC1.Tg Mice Conferred Protection in Adoptive Transfer Experiments (Winn Assay). **(A)** The sorted spleen-derived effector T-cells from MUC1.Tg mice immunized with rotating tumor cell lysates were co-injected with B16.MUC1 tumor cells (T-cell to tumor cell ratio of 10:1) either directly or after stimulation *in vitro* with DCs pulsed with B16.MUC1 tumor cell lysates. T-cells from non-immunized MUC1.Tg mice were co-injected with B16.MUC1 tumor cells as controls. The mice that received T-cells after *in vitro* sensitization showed complete protection from tumor growth, (p<0.001). **(B)** The B16.MUC1 tumors resistant to T-cells from immunized MUC1.Tg mice showed low MUC1 expression vs non-immunized mice (p = 0.02). **(C)** Corresponding histograms of MUC1 expression are shown. Experiment was repeated two times, n = 4 mice/group.

As shown above in Fig 6A, peptide plus CpG vaccination was rendered therapeutically effective if it was continued into the tumor-bearing state; the absence of MUC1-sensitized T-cells, or a lack of concurrent tumor and vaccine, negated therapy. The need for an antigen-specific re-stimulation in the presence of tumor was validated by Winn assays. Inoculation of B16.MUC1 with either naive T-cells or lysate-immunized MUC1-specific T-cells did not prevent tumor growth (Fig 7A). In contrast, the same mixture of lysate-immunized T-cells following an *in vitro* stimulation with B16.MUC1 lysate-pulsed DCs resulted in consistent cure (p<0.001). Even though tumor growth was not prevented by the lysate-immunized T-cells in the absence of DCs, the emerging tumors showed down regulation of MUC1 as compared to naive T-cells, indicating there was a residual memory response that applied a degree of selection pressure (Fig 7B and 7C)

### Immunologic Memory is Manifest as Recurrent Immunoediting in Tumor-Rejecting MUC1.Tg Mice

**Fig 8:**
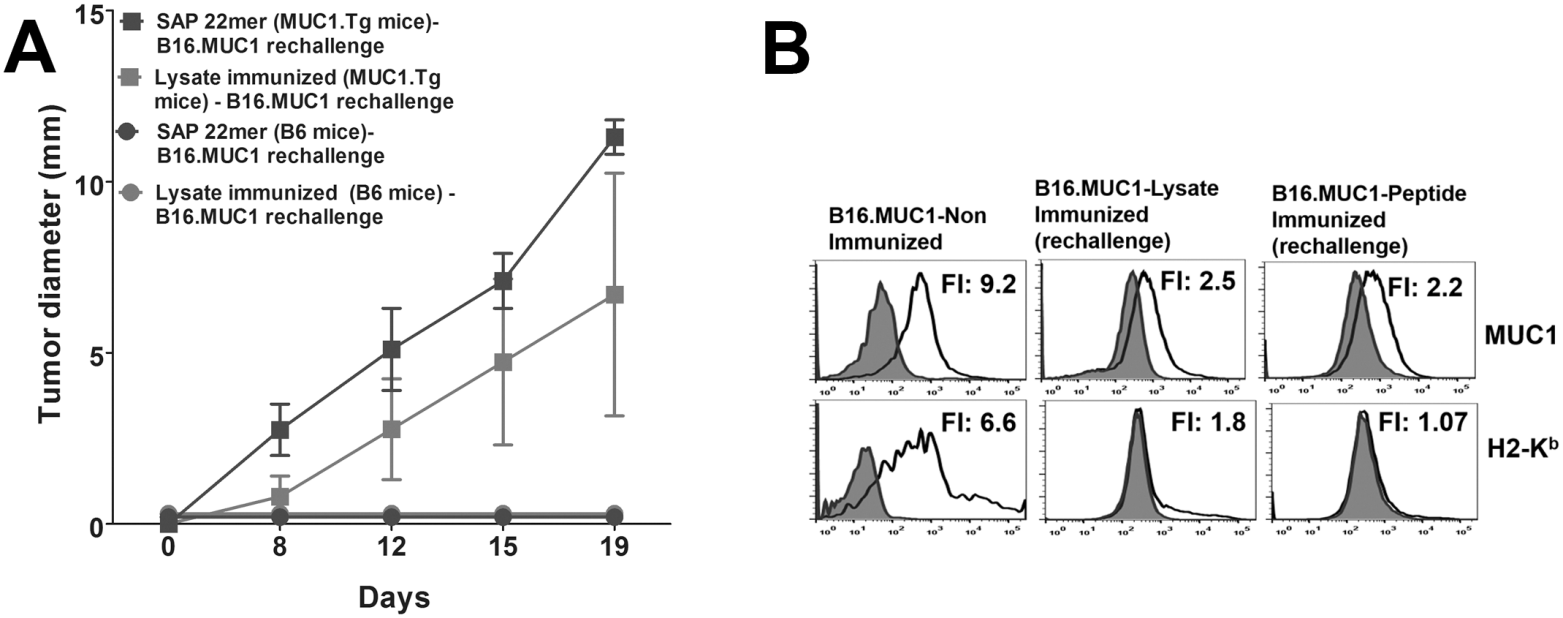
MUC1.Tg Mice Exhibited Incomplete Immunological Memory Following Tumor Rechallenge. **(A)** Mice that rejected the primary tumor challenge (previously immunized with SAP 22mer or rotating cell lysates) were re-challenged with B16.MUC1 from 75 to 200 days later. There was no further vaccination following the re-challenge. WT mice consistently rejected the tumor whereas MUC1.Tg mice displayed tumor outgrowth that **(B)** down regulated MUC1 and class I expression. n = 2 for WT mice/group or 5 MUC1.Tg mice/group.

We investigated whether the rejection of MUC1-expressing tumors by vaccine-sensitized T-cells resulted in sustained immunologic memory. Both WT and MUC1.Tg mice that rejected the primary tumor challenge were rechallenged with B16.MUC1 from 75 to 200 days thereafter. After the first rejection, mice received no further vaccinations. We hypothesized that incomplete immunological memory would be evidenced by outgrowth of tumor with down regulated MUC1 and MHC molecules. We observed that rechallenged WT mice consistently rejected tumor rechallenge (Fig 8A), whereas rechallenged MUC1.Tg mice displayed outgrowth of B16 tumors that down regulated MUC1 and MHC expression (Fig 8B). Therefore, re-vaccination may be required when self-antigen is the catalyst of tumor rejection.

## Discussion

The tumor-associated antigen MUC1 is over-expressed by about three-fourths of all lethal human cancers, and was recently ranked number two by the NCI among all known tumor-associated antigens for meriting fast-tracked clinical prioritization [48]. The present report identifies several determinants of successful MUC1 immunotherapy which are consistently observed in clinically relevant MUC1.Tg mouse models.

We and others have confirmed that tolerance of MUC1.Tg mice to MUC1 can be reversed by vaccination [29,30,49,50]. The data reported presently demonstrate unequivocal expansion of both CD4^+^ and CD8^+^ T1-type (IFN-γ producing) MUC1-specific responses in vaccinated MUC1.Tg mice. Even though background MUC1 expression in MUC1.Tg mice naturally skews the anti-MUC1 T-cell repertoire compared to WT mice, there were no discernible differences in generated T-cell avidity (S1 Fig) [45]. Thus, consistent with previous reports, we did not observe evidence for thymic deletion of high affinity responses, suggesting that MUC1 tolerance is largely a reversible peripheral phenomenon [51]. Furthermore, immunotargeting MUC1 could be used to achieve major therapeutic effects without engendering detectable autoimmune toxicity.

It was possible to use vaccination to expand T-cell responses against all tested MUC1 regions including TR, degenerate TR sequences, cytoplasmic tail, and signal sequence. In addition, as also reported for several other antigens [52,53,54,55], profound intramolecular epitope spreading was observed during vaccination, with, for example, targeting of the TR leading to cross-recognition of the CT and vice versa. Such intramolecular spreading is attributable to the known background autoantigen expression of MUC1 protein in the transgenic animals. Remarkably, intramolecular spreading did not occur when a sham CT peptide with only a single additional amino acid was used as the immunogen (not shown). This illustrates how precisely the immune system can regulate expansion of the repertoire.

As a consequence of such intramolecular spreading and/or intrinsically promiscuous cross-recognition, CD4^+^ and CD8^+^ T-cell recognition of both glycosylated and non-glycosylated MUC1 epitopes was an invariable consequence of vaccination with any of the antigen forms tested. This was true even when MUC1 priming consisted of only 2 non-glycosylated 9mers. Furthermore, even when there was exposure only to synthetic peptide during vaccination, the T-cell repertoire naturally encompassed recognition of tumor-associated MUC1 [6], evidenced by reactivity to multiple, whole-cell tumor digests, and protection against *in vivo* tumor challenges, particularly when vaccination was continued post tumor challenge (Fig 6A). These results suggest that natural diversification of the anti-MUC1 immune response during vaccination may obviate the need to custom-vaccinate against individual tumor glycosylation variants which can arise unpredictably even among patients with the same cancer type [12,56].

Nonetheless, despite the ability of every tested vaccine to generate a diverse anti-MUC1 response, striking differences were evident in resultant anti-MUC1 T-cell repertoires which may account for differences in therapeutic potency. The most therapeutically effective vaccine immunogen, rotating lysates, resulted in a distinctively prominent T-cell sub-repertoire exclusively recognizing glycosylated epitopes, whereas the least effective immunogen, APG 22mer, especially promoted exclusive recognition of non-glycosylated epitopes (Fig 2A and 2B). Furthermore, rotating lysates proved more consistently effective than long peptides for promoting T-cell responses against embedded peptide sequences. For example, due to likely cleavage restraints [46], vaccination to the long peptide APG 22mer resulted in T-cell recognition of the embedded APGSTAPPA but not the embedded SAPDTRPAP (Fig 2A). In contrast, no cleavage restrictions were evident when lysates were employed, even though all MUC1 peptides in tumor lysate are intrinsically embedded. In fact, vaccination with rotating lysates resulted in T-cell recognition of every tested peptide, whether long or short (Fig 2C). Similarly, only vaccination with rotating lysates resulted in T-cell recognition of every tested syngeneic MUC1^+^ tumor digest (Fig 3E).

It is important to note, however, that the ability of T-cells to have a therapeutic effect occasionally did not correlate to their reactivity with tumor digests *in vitro* (Fig 3E): for example, vaccination with the SAP 22mer resulted in T-cells that did not recognize B16.MUC1 digests (Fig 3E), yet vaccinated animals were able to control B16.MUC1 tumor growth (Fig 6A). In contrast, APG 22mer vaccination resulted in strong recognition of B16.MUC1 digests, yet such T-cells were ineffective therapeutically against B16.MUC1. Such seeming disparities may simply reflect the fact that digests come from mice that were not vaccinated, hence not reflective of each model’s dynamic expression of MUC1 when subjected to T-cell selection pressures.

When vaccination was confined to the period prior to tumor challenge, individual antigen preparations varied in their protective efficacy, yet even vaccines which failed to prevent tumor progression consistently resulted in selection pressure, provoking down regulation of tumor MUC1 as well as MHC molecules (Fig 5). Immunotherapy targeting other antigens such as HER2 and MART-I has also shown evidence of immunoediting and tumor escape [57,58,59].

Greater efficacy was achieved by continuing vaccination into the tumor-bearing period, demonstrating the T-cells’ requirement for stronger re-stimulation than that provided by MUC1-expressing tumors alone. Winn assays [60] further demonstrated that lysate-sensitized T-cells from hyperimmunized MUC1.Tg mice were as ineffective as naïve T-cells for controlling directly admixed MUC1^+^ tumor challenges, even though the MUC1-hyperimmune T-cells provoked down regulation of tumor MUC1 and H-2K^b^ (Fig 7B and 7C). In contrast, *in vitro* re-stimulation of the MUC1-hyperimmune T-cells with B16.MUC1 lysate-pulsed DCs prior to the Winn assay rendered them therapeutically active and prevented tumor outgrowth. These results confirm previous findings [61] that even large numbers of immune T cells could not mediate tumor regression without being in a correct state of activation [62]. Incorporating surrogate T-cell activation outside the tumor bed, either additional vaccination, checkpoint inhibitors and/or *ex vivo* T-cell activation, are rational clinical strategies to overcome tumor escape.

## Conclusions

In conclusion, vaccinations readily reversed MUC1 tolerance in MUC1 transgenic mice and favored emergence of high avidity T1-type T-cell responses which were indistinguishable from wildtype mice, all without detected autoimmune toxicity. Immunoediting of MUC1 expression on tumors was invariably observed in every protection failure, suggesting an activated immune system. The combination of a vaccine together with the timely administration of checkpoint inhibitors could result in effective therapy for many types of tumors judged to be non-immunogenic and hence, not responsive to PD-1/PD-L1 axis blockade. These studies are under investigation presently. A key observation was that vaccination even with minimalist antigens such as non-glycosylated 9mers produced CD8^+^ as well as CD4^+^ T-cell repertoires that recognized both non-glycosylated and glycosylated peptides as well as tumor-associated MUC1. This pan recognition is of prime importance given that MUC1 is a heavily glycosylated protein. The diversification of the immune response and the intramolecular epitope spreading may obviate the need for development of custom vaccines for different glycoforms of MUC1 which arise among patients with tumors originating from different tissues or even within the same tumor type. As MUC1 is aberrantly expressed on about 75% of lethal human tumors, effective vaccination strategies will have widespread applicability, especially as an adjunct to current immunomodulatory therapies such as checkpoint inhibitors.

## Materials and Methods

### Peptide Synthesis

The MUC1 peptides used in the study (Fig 1) were synthesized either at the Complex Carbohydrate Research Centre, University of Georgia or at the Mayo Clinic Protein Synthesis Core laboratory, Rochester, MN as described previously [30]: Tn is αGalNAc *O*-linked to serine/threonine. Peptides were chosen incorporating either the H-2K^b^ (SAPDTRPAP) or D^b^ (APGSTAPPA) epitope at the beginning sequence. Purified CpG ODN 1826 (CpG, Coley Pharmaceuticals) was reconstituted in sterile pyrogen-free water at a concentration of 3.3 μg/μl and stored at -80°C for future use.

### Reagents

Culture media (CM) for dendritic cells (DCs) and T-cells was RPMI 1640 (Invitrogen) supplemented with 10% heat deactivated FBS (Gibco) or 1% mouse serum (MS), 200mM L-glutamine (Lonza), 100mM sodium pyruvate (Lonza), 50mM 2-mercaptoethanol (Gibco), 0.25% penicillin/streptomycin (Lonza), non-essential amino acids (1%v/v), gentamicin (12.5ug/ml) (Lonza) and amphotericin B (12.5ng/ml) (Lonza). All tumor cell lines were maintained in DMEM (Invitrogen) supplemented with 10% FBS (Gemini), 1% glutamax (Invitrogen) and 1% penicillin/streptomycin. G418 (Adipogen) was used for maintaining neo and MUC1 transfected cell lines.

### Cell Lines and Mice

Cell lines are as follows: mammary gland: C57mg, C57mg.MUC1 [36]; colon: MC38.neo (generous gift from J. Schlom), MC38.MUC1 (generous gift from D. Kufe) [49]; melanoma: B16.neo, B16.MUC1 [41]; lymphoma: EL4.neo, EL4.MUC1 [63]; pancreas: Panc02.neo, Panc02.MUC1 [64] (generous gifts from M.A. Hollingsworth), and KCKO and KCM (generous gift from P Mukherjee) [65]. KCM and KCKO were generated from spontaneous tumors in the MUC1.Tg (KCM) or Muc1^-/-^ mice (KCKO) mated with the Kras^LSL-G12D^ mice (all on the C57BL/6 background). All MUC1-expressing cell lines have full length *MUC1*. Transfected cell lines were maintained in DMEM complete medium either with G418, 150ug/ml (Panc02.neo, Panc02.MUC1, EL4neo, EL4.MUC1, MC38.neo, MC38.MUC1 and C57mg.MUC1) or with G418, 300ug/ml (B16.neo and B16.MUC1 cell lines). Cell lines represent commonly detected tumor types, including those known to be difficult to cure such as melanoma, breast and pancreas lines. All cell lines were verified at the end of the experiments as being entirely of mouse origin from the C57BL/6 strain and no mammalian interspecies contamination was detected (IDEXX BioResearch, Columbia, MO). All cells were determined to be free of mycoplasma (IDEXX).

C57BL/6 mice, (8 to 12-weeks, Jackson Laboratory) and MUC1.Tg mice (bred in house, also available from Jackson Laboratory) [41] were used for immunizations and for generation of tumors *in vivo*. All mice are on the C57BL/6 strain.

### Mouse Husbandry and Ethics Statement

This study was carried out in strict accordance with the recommendations in the Guide for the Care and Use of Laboratory Animals. The protocol was approved by the Institutional Animal Care and Use Committee of the Mayo Clinic (A42414). All mice fell into pain category A on the IACUC form as not needing analgesics or anaesthetics. All efforts were made to minimize suffering. Mice were examined for failure to eat or drink, for ruffled fur or hunched posture and for failure to move freely about the cage. These mice would have been promptly euthanized by CO_2_ inhalation at the onset of any such symptoms. Six mice died after the time of vaccination but prior to the time point of selection into each experiment and were excluded from all analyses. Cause of death was unknown (found dead in cage) and these cases, despite small numbers, did not appear to be associated with any specific vaccine.

Mice that had received injections of tumor cells were examined twice weekly until tumors formed. Mice bearing tumors were palpated every two days until sacrifice. Mice that were immunized with SAP 22mer or lysate and did not form tumors were examined twice weekly until rechallenged with tumor at 70 to 200 days after the first tumor challenge as shown in Fig 8. Other surviving mice were monitored twice weekly until sacrifice by inhalation of CO_2_ at about 150 days following last tumor injection. Per IACUC regulations, all surviving mice with tumors were sacrificed when tumors reached 10% of body weight, were ulcerated, or reached 14 x 14 mm^2^. Sacrifice was by inhalation of CO_2_ as per IACUC regulations. Our AAALAC Accreditation Number is 000880 and the most recent date of accreditation was 06/26/2013. Our OLAW Assurance Number is A3291-01.

### Tumor Lysate Preparation

To generate tumor cell lysates, MUC1-expressing cells (C57mg.MUC1; KCM; EL4.MUC1; B16.MUC1; Panc02.MUC1) (5x10^7^) were resuspended in 1 ml of PBS and lysed by 5 cycles of freezing in liquid nitrogen and thawing at 37°C in a water bath. The cell lysates were stored at -80°C until use.

### Tumor Digest

The tumor tissues were digested enzymatically and irradiated with 10,000 cGy prior to freezing at -80°C as described previously [66].

### Peptide Vaccination

MUC1.Tg or WT mice were given two immunizations at one week intervals subcutaneously (sc) at the base of the tail. Vaccine consists of MUC1 peptide (100 μg for first immunization or 50ug for subsequent immunizations) and CpG (50 μg), all emulsified in IFA (DIFCO). For therapeutic studies, mice were vaccinated at 10 day intervals 3 times prior to tumor challenge or with 2 immunizations prior to tumor challenge followed by 2 more immunizations after tumor challenge.

### Rotating Lysate Vaccination

MUC1.Tg or WT mice were vaccinated sc every three weeks with MUC1-expressing cell lysates (1^st^ immunization—C57mg.MUC1; 2^nd^—KCM; 3^rd^—EL4.MUC1; 4^th^—C57mg.MUC1) before tumor implantation. For each treatment, tumor cell lysate equivalent to 5 x 10^6^ cells was mixed with 50 ug of CpG and IFA for a total volume of 200 μl and injected sc on both sides at the base of the tail.

### DC and T-Cell Culture

DCs were generated *in vitro* as described [66]. The L-selectin CD62^low^ fraction of T-cells (effector) was isolated from lymph nodes and spleens of immunized mice. The isolated cells (2 x 10^6^/ml) were activated with antigen-pulsed DCs at T-cell to DC ratio of 8:1. Two days later, the T-cells were split (1:2) with CM, 1% MS, rhIL-2, 24 IU (4 Cetus units)/ml (Chiron); rhIL-7, 50 ng/ml (Miltenyi Biotech); and rhIL-15, 5 ng/ml (Peprotech). The cells were further stimulated with rhIL-2, 24 IU (4 Cetus units)/ml on days 5, 8 and 11. The (IFN-γ) ICC assay was performed on days 7 or 14.

### Intracellular Cytokine Interferon-γ Assays (ICC)

Cultured T-cells (1× 10^6^/ml) were restimulated with either DCs pulsed with peptides (5x10^4^/well) (20:1) or whole-cell irradiated tumor digests (1× 10^6^/well) (1:1). After 4 hrs of co-culture, monensin (0.7μl/ well) (BD Pharmingen) was added and co-culture continued for another 12 hours. Stimulated T-cells were FcR blocked and stained with FITC anti-CD4 (clone: GK1.5, BD Pharmingen) and APC anti-CD8 (clone: 53–6.7, eBioscience). Following fixation/permeabilization, cells were stained with PE anti-mouse IFN-γ (BD Pharmingen) and analyzed by flow cytometry on LSR Fortessa Flow Cytometer (BD Biosciences) using DIVA software. Antibody amounts used for staining were according to manufacturer’s specifications.

### Tumor Generation

MUC1.Tg or WT mice were injected either ten days (peptide vaccine) or three weeks (lysate vaccine) after the last immunization sc in the left flank with cancer cells (B16.MUC1-5x10^5^ cells; Panc02.MUC1-1x10^6^ cells; MC38.MUC1-5x10^5^ cells) in 100μl of PBS. Tumor cell lines showed strong expression of MUC1 as determined by flow cytometry with anti-MUC1 antibody CD227 (Clone HMPV, BD Pharmingen) (Fig 3A). Mice were palpated every two days until sacrifice. Per IACUC regulations, all surviving mice were sacrificed when tumors reached 10% of body weight, were ulcerated or reached 14 x 14 mm^2^.

### Tumor Digest Analysis

Tumors were digested as described above, washed with staining buffer (0.5% FBS in PBS), FC blocked and stained for surface expression (CD45APC/Cy7, clone:30-F11, eBioscience; CD11bAPC, clone:M1/70, eBioscience; CD11cBV570, clone-N418, Biolegend; H-2KbPE, Clone-AFb-88.5, BD Pharmingen; I-Ab-PE, Clone AF6-120.1, BD Pharmingen; CD227FITC (MUC1)). Antibody amounts used for staining were according to manufacturer’s specifications. Fluorescent index was calculated as specific geometric mean/isotype geometric mean. LSR Fortessa flow cytometer was used for multiparameter flow cytometry of stained cells. FlowJo software (Tree star) and Diva software were used for analyses.

### Winn assay

The Winn assay was performed as described previously [60]. Briefly, the T-cells from experimental groups were mixed with B16.MUC1 tumor cells (10:1; 2x10^6^ T-cells: 2x10^5^ tumor cells) and inoculated into recipient (non-irradiated) mice. T-cells from spleen were prepared by negative selection using Dynabeads (Invitrogen).

### DC Presentation of MUC1

On day 8 DCs were incubated with MUC1 peptides at a concentration of 10μg/ml. After overnight stimulation the antigen pulsed and unpulsed DCs were further matured with LPS (1ug/ml) for 2 hrs. The cells were then harvested and stained for the surface expression of CD11c, H-2K^b^, I-A^b^ and MUC1 epitope (BC2 Alexa 488 (epitope:APDTR) [47] and B27.29 (epitope:PDTRP)) [67] and analyzed by flow cytometry.

### Confocal Microscopy

As described above, rhFLT3-L and IL-6-activated BM cells were transferred to polylysine-coated glass wells with 10 ng/ml rmGM-CSF, 10 ng/ml rmIL-4. After 24 hrs culture, the DCs were pulsed with the peptides and stimulated with LPS. The cells were washed, fixed for 2 min in ethanol (-20°C) and blocked overnight with 1% BSA (Sigma) in PBS, washed and stained for 1hr with anti-MUC1 (BC2-Alexa488) and anti H-2K^b^ (AF6-88.5, BD Pharmingen), followed by a secondary goat anti-mouse IgGAlexa633 (Invitrogen) at 1:100 dilution for 20 min at room temperature, washed and mounted with Prolong Gold Antifade Reagent (Invitrogen). Imaging was performed on a Zeiss laser scanning microscope with a 63X 1.4 NA oil DIC immersion objective and analyzed using Zeiss LSM image browser.

### Statistical Analysis

A mixed model with fixed effects of group and time was used to evaluate tumor diameter (mm). The interaction effect of group x time was examined for group comparisons for each study experiment. When significant, post-hoc comparisons for individual time points were compared between groups using Student’s t-test. A p value <0.05 was considered statistically significant. SAS version 9.3 (Cary, NC) was used for analysis.

## Supporting Information

S1 Fig High Avidity MUC1-Specific T-Cells were Generated from both MUC1.Tg and WT Mice. MUC1.Tg and WT mice were given three immunizations with vaccine containing APG 22mer. Lymph node-derived T-cells were culture expanded with DCs unpulsed or pulsed with the immunizing peptide in varying concentrations for 14 days in the presence or absence of IL-12 at 2 ng/ml. Antigen-specific T-cells were enumerated for intracellular IFN-γ production when re-stimulated with DCs pulsed with APG 22mer. A representative of 3 experiments is shown; pools of 7 mice were used.
(TIF)
