# Supplementary figures and images for: MUC1 Vaccines, Comprised of Glycosylated or Non-Glycosylated Peptides or Tumor-Derived MUC1, Can Circumvent Immunoediting to Control Tumor Growth in MUC1 Transgenic Mice
Source: PLoS One. 2016 Jan 20;11(1):e0145920. doi: 10.1371/journal.pone.0145920 (PMC4720451; doi:10.1371/journal.pone.0145920)

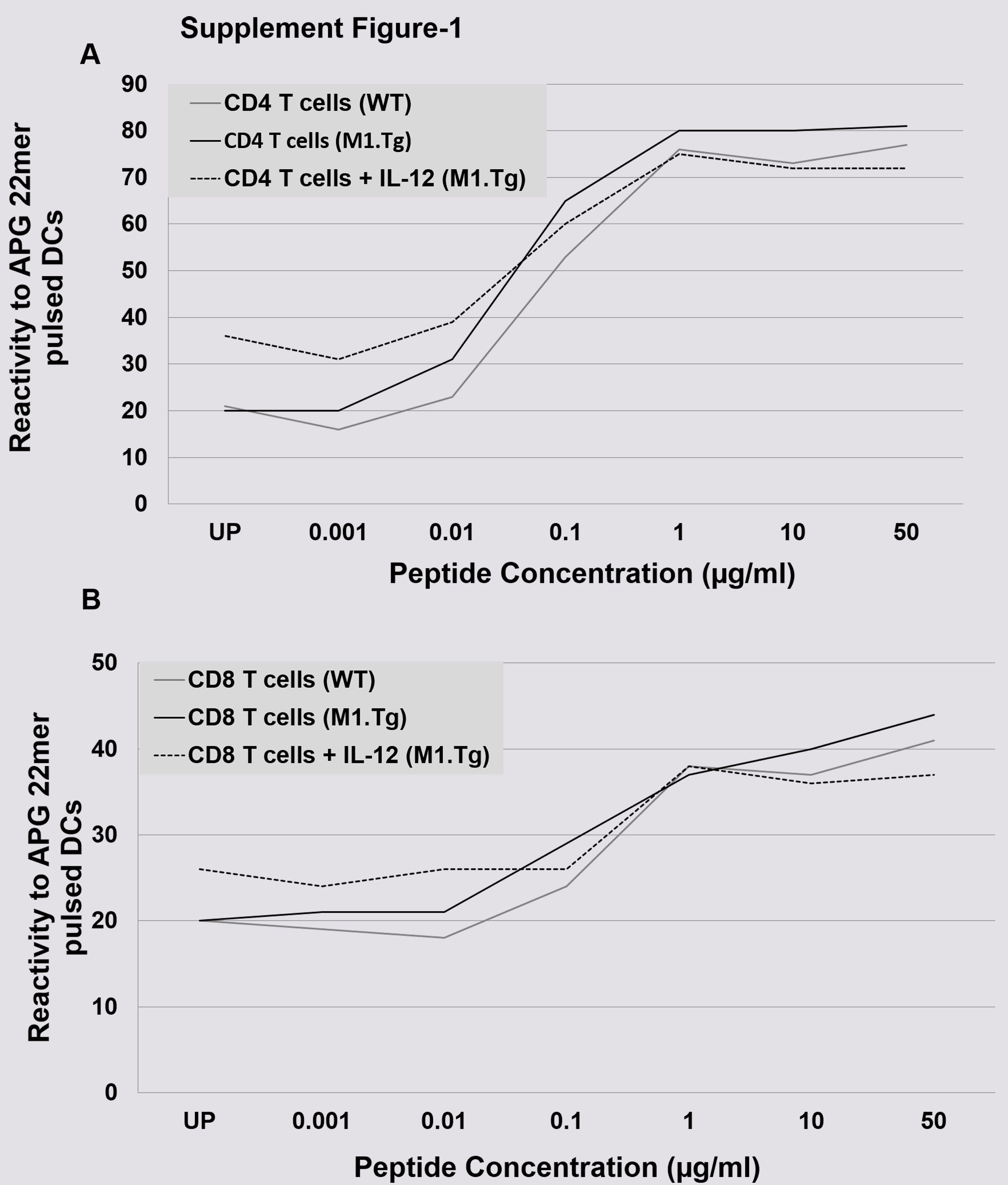

Supplement: S1 Fig — MUC1.Tg and WT mice were given three immunizations with vaccine containing APG 22mer. Lymph node-derived T-cells were culture expanded with DCs unpulsed or pulsed with the immunizing peptide in varying concentrations for 14 days in the presence or absence of IL-12 at 2 ng/ml. Antigen-specific T-cells were enumerated for intracellular IFN-γ production when re-stimulated with DCs pulsed with APG 22mer. A representative of 3 experiments is shown; pools of 7 mice were used. (TIF) [file pone.0145920.s001.tif]
